# Supplementary material for: Efficiency of Calcium Fructoborate-Loaded Novel Natural Niosomes Compared to Traditional Liposomes and Niosomes in Rat Ischemia–Reperfusion Injury Model
Source: Pharmaceutics. 2025 Nov 6;17(11):1434. doi: 10.3390/pharmaceutics17111434 (PMC12655740; doi:10.3390/pharmaceutics17111434)

TÜRKİYE ENERJİ, NÜKLEER VE MADEN ARAŞTIRMA KURUMU
Bor Araştırma Enstitüsü (BOREN)

TENMAK

Dumlupınar Bulvarı, No:166 D-Blok 06530 Çankaya-ANKARA

TESTING REPORT

2025.07.000328
07.2025

Customer name/Address	:	İstanbul Kent Üniversitesi/Cihangir mah. sıraselviler cad. no:71 Beyoğlu İSTANBUL
Order Number	:	2025.07.000328
Name and Identity of Test Item	:	Doğal Niozomal Kalsiyum Fruktoborat, Niozomal Kalsiyum Fruktoborat, Lipozomal Kalsiyum Fruktoborat, Standart Kalsiyum Fruktoborat
The Date of Receipt of Test Item	:	30.06.2025
Remarks	:	-
Date of Test	:	10.07.2025 – 14.07.2025
Number of Pages of the Report	:	6

The test and/or measurement results, the uncertainties (if applicable) with confidence probability and test methods are given on the following pages which are part of this report

Seal	Date	Analyst	Approval/Date
TENMAK<br>Bor Araştırma Enstitüsü<br>Dumlupınar Bulvarı<br>(Eskişehir Yolu 7.km.) No:166 D Blok<br>(06560) Çankaya/ANKARA	21.07.2025	Merve ÖZTÜRK	Sezgin ÖZKASAPOĞLU<br>21.07.2025

This report shall not be partially copied or reproduced without written consent of the laboratory. Reports without signature and seal are invalid

Tel: +90 (312) 201 36 00 *Faks:* +90 (312) 219 80 55 boren.tenmak.gov.tr numune@boren.gov.tr

TENMAK

TÜRKİYE ENERJİ, NÜKLEER VE MADEN
ARAŞTIRMA KURUMU
Bor Araştırma Enstitüsü (BOREN)

Dumlupınar Bulvarı, No:166 D-Blok 06530 Çankaya - ANKARA

Analysis results of the sample coded as Doğal Niozomal Kalsiyum Fruktoborat

Parameter	Unit	Results	Testing Methods	Environmental Conditions
B	mg/L	169,355	Modified from EPA Methods 3051A and 6010D	Average ambient temperature and humidity at the time of analysis 25,5 °C %35,5
Ca	mg/L	279,956	Modified from EPA Methods 3051A and 6010D	Average ambient temperature and humidity at the time of analysis 25,5 °C %35,5
Fe	mg/L	<0.010	Modified from EPA Methods 3051A and 6010D	Average ambient temperature and humidity at the time of analysis 25,5 °C %35,5
K	mg/L	63,691	Modified from EPA Methods 3051A and 6010D	Average ambient temperature and humidity at the time of analysis 25,5 °C %35,5
As	mg/L	0,009	Modified from EPA Methods 3051A and 6010D	Average ambient temperature and humidity at the time of analysis 25,5 °C %35,5
Be	mg/L	0,002	Modified from EPA Methods 3051A and 6010D	Average ambient temperature and humidity at the time of analysis 25,5 °C %35,5
Ba	mg/L	0,039	Modified from EPA Methods 3051A and 6010D	Average ambient temperature and humidity at the time of analysis 25,5 °C %35,5
Cr	mg/L	0,011	Modified from EPA Methods 3051A and 6010D	Average ambient temperature and humidity at the time of analysis 25,5 °C %35,5
Co	mg/L	0,003	Modified from EPA Methods 3051A and 6010D	Average ambient temperature and humidity at the time of analysis 25,5 °C %35,5
Cd	mg/L	<0.001	Modified from EPA Methods 3051A and 6010D	Average ambient temperature and humidity at the time of analysis 25,5 °C %35,5
Cu	mg/L	0,060	Modified from EPA Methods 3051A and 6010D	Average ambient temperature and humidity at the time of analysis 25,5 °C %35,5
Pb	mg/L	<0.001	Modified from EPA Methods 3051A and 6010D	Average ambient temperature and humidity at the time of analysis 25,5 °C %35,5
Mn	mg/L	0,064	Modified from EPA Methods 3051A and 6010D	Average ambient temperature and humidity at the time of analysis 25,5 °C %35,5

Tel: +90 (312) 201 36 00 *Faks:* +90 (312) 219 80 55 boren.tenmak.gov.tr numune@boren.gov.tr

Ni	mg/L	0,005	Modified from EPA Methods 3051A and 6010D	Average ambient temperature and humidity at the time of analysis 25,5 °C %35,5
Zn	mg/L	0,084	Modified from EPA Methods 3051A and 6010D	Average ambient temperature and humidity at the time of analysis 25,5 °C %35,5

Analyzes marked with (*) in the report are accredited.

Analysis results of the sample coded as Niozomal Kalsiyum Fruktoborat

Parameter	Unit	Results	Testing Methods	Environmental Conditions
B	mg/L	177,607	Modified from EPA Methods 3051A and 6010D	Average ambient temperature and humidity at the time of analysis 25,5 °C %35,5
Ca	mg/L	301,964	Modified from EPA Methods 3051A and 6010D	Average ambient temperature and humidity at the time of analysis 25,5 °C %35,5
Fe	mg/L	<0.010	Modified from EPA Methods 3051A and 6010D	Average ambient temperature and humidity at the time of analysis 25,5 °C %35,5
K	mg/L	2,732	Modified from EPA Methods 3051A and 6010D	Average ambient temperature and humidity at the time of analysis 25,5 °C %35,5
As	mg/L	0,010	Modified from EPA Methods 3051A and 6010D	Average ambient temperature and humidity at the time of analysis 25,5 °C %35,5
Be	mg/L	0,003	Modified from EPA Methods 3051A and 6010D	Average ambient temperature and humidity at the time of analysis 25,5 °C %35,5
Ba	mg/L	<0.001	Modified from EPA Methods 3051A and 6010D	Average ambient temperature and humidity at the time of analysis 25,5 °C %35,5
Cr	mg/L	0,015	Modified from EPA Methods 3051A and 6010D	Average ambient temperature and humidity at the time of analysis 25,5 °C %35,5
Co	mg/L	0,001	Modified from EPA Methods 3051A and 6010D	Average ambient temperature and humidity at the time of analysis 25,5 °C %35,5
Cd	mg/L	<0.001	Modified from EPA Methods 3051A and 6010D	Average ambient temperature and humidity at the time of analysis 25,5 °C %35,5
Cu	mg/L	0,035	Modified from EPA Methods 3051A and 6010D	Average ambient temperature and humidity at the time of analysis 25,5 °C %35,5
Pb	mg/L	0,003	Modified from EPA Methods 3051A and 6010D	Average ambient temperature and humidity at the time of analysis 25,5 °C %35,5

Mn	mg/L	0,014	Modified from EPA Methods 3051A and 6010D	Average ambient temperature and humidity at the time of analysis 25,5 °C %35,5
Ni	mg/L	<0.001	Modified from EPA Methods 3051A and 6010D	Average ambient temperature and humidity at the time of analysis 25,5 °C %35,5
Zn	mg/L	0,089	Modified from EPA Methods 3051A and 6010D	Average ambient temperature and humidity at the time of analysis 25,5 °C %35,5

Analyzes marked with (*) in the report are accredited.

Analysis results of the sample coded as Lipozomal Kalsiyum Fruktoborat

Parameter	Unit	Results	Testing Methods	Environmental Conditions
B	mg/L	157,689	Modified from EPA Methods 3051A and 6010D	Average ambient temperature and humidity at the time of analysis 25,5 °C %35,5
Ca	mg/L	292,766	Modified from EPA Methods 3051A and 6010D	Average ambient temperature and humidity at the time of analysis 25,5 °C %35,5
Fe	mg/L	<0.010	Modified from EPA Methods 3051A and 6010D	Average ambient temperature and humidity at the time of analysis 25,5 °C %35,5
K	mg/L	1,127	Modified from EPA Methods 3051A and 6010D	Average ambient temperature and humidity at the time of analysis 25,5 °C %35,5
As	mg/L	0,013	Modified from EPA Methods 3051A and 6010D	Average ambient temperature and humidity at the time of analysis 25,5 °C %35,5
Be	mg/L	0,002	Modified from EPA Methods 3051A and 6010D	Average ambient temperature and humidity at the time of analysis 25,5 °C %35,5
Ba	mg/L	<0.001	Modified from EPA Methods 3051A and 6010D	Average ambient temperature and humidity at the time of analysis 25,5 °C %35,5
Cr	mg/L	0,015	Modified from EPA Methods 3051A and 6010D	Average ambient temperature and humidity at the time of analysis 25,5 °C %35,5
Co	mg/L	0,003	Modified from EPA Methods 3051A and 6010D	Average ambient temperature and humidity at the time of analysis 25,5 °C %35,5
Cd	mg/L	<0.001	Modified from EPA Methods 3051A and 6010D	Average ambient temperature and humidity at the time of analysis 25,5 °C %35,5
Cu	mg/L	0,021	Modified from EPA Methods 3051A and 6010D	Average ambient temperature and humidity at the time of analysis 25,5 °C %35,5

PR.31-F.02 Rev.00/08.09.2021
Tel: +90 (312) 201 36 00 *Faks:* +90 (312) 219 80 55 boren.tenmak.gov.tr numune@boren.gov.tr

Pb	mg/L	0,001	Modified from EPA Methods 3051A and 6010D	Average ambient temperature and humidity at the time of analysis 25,5 °C %35,5
Mn	mg/L	0,017	Modified from EPA Methods 3051A and 6010D	Average ambient temperature and humidity at the time of analysis 25,5 °C %35,5
Ni	mg/L	<0.001	Modified from EPA Methods 3051A and 6010D	Average ambient temperature and humidity at the time of analysis 25,5 °C %35,5
Zn	mg/L	0,082	Modified from EPA Methods 3051A and 6010D	Average ambient temperature and humidity at the time of analysis 25,5 °C %35,5

Analyzes marked with (*) in the report are accredited.

Analysis results of the sample coded as Standart Kalsiyum Fruktoborat

Parameter	Unit	Results	Testing Methods	Environmental Conditions
B	mg/L	161,662	Modified from EPA Methods 3051A and 6010D	Average ambient temperature and humidity at the time of analysis 25,5 °C %35,5
Ca	mg/L	270,140	Modified from EPA Methods 3051A and 6010D	Average ambient temperature and humidity at the time of analysis 25,5 °C %35,5
Fe	mg/L	<0.010	Modified from EPA Methods 3051A and 6010D	Average ambient temperature and humidity at the time of analysis 25,5 °C %35,5
K	mg/L	0,184	Modified from EPA Methods 3051A and 6010D	Average ambient temperature and humidity at the time of analysis 25,5 °C %35,5
As	mg/L	0,002	Modified from EPA Methods 3051A and 6010D	Average ambient temperature and humidity at the time of analysis 25,5 °C %35,5
Be	mg/L	0,001	Modified from EPA Methods 3051A and 6010D	Average ambient temperature and humidity at the time of analysis 25,5 °C %35,5
Ba	mg/L	<0.001	Modified from EPA Methods 3051A and 6010D	Average ambient temperature and humidity at the time of analysis 25,5 °C %35,5
Cr	mg/L	0,006	Modified from EPA Methods 3051A and 6010D	Average ambient temperature and humidity at the time of analysis 25,5 °C %35,5
Co	mg/L	<0.001	Modified from EPA Methods 3051A and 6010D	Average ambient temperature and humidity at the time of analysis 25,5 °C %35,5
Cd	mg/L	<0.001	Modified from EPA Methods 3051A and 6010D	Average ambient temperature and humidity at the time of analysis 25,5 °C %35,5

Cu	mg/L	0,006	Modified from EPA Methods 3051A and 6010D	Average ambient temperature and humidity at the time of analysis 25,5 °C %35,5
Pb	mg/L	0,008	Modified from EPA Methods 3051A and 6010D	Average ambient temperature and humidity at the time of analysis 25,5 °C %35,5
Mn	mg/L	0,010	Modified from EPA Methods 3051A and 6010D	Average ambient temperature and humidity at the time of analysis 25,5 °C %35,5
Ni	mg/L	0,008	Modified from EPA Methods 3051A and 6010D	Average ambient temperature and humidity at the time of analysis 25,5 °C %35,5
Zn	mg/L	0,019	Modified from EPA Methods 3051A and 6010D	Average ambient temperature and humidity at the time of analysis 25,5 °C %35,5

Analyzes marked with (*) in the report are accredited.

Disclaimer Description:

Conformity with Requirements or Specifications:

Definition of Decision Rule:

Specification, Standard, Regulatory Information Requesting a Declaration of Conformity:

The following information will be filled when the Decision Rule is applied:

The Results Applied Decision Rule	Limit Values of Legislation or Specification	Evaluation of the State of Meeting the Limit Values of the Analysis Results
		☐ Pass ☐ Fail
		☐ Pass ☐ Fail

Statement that the results are valid for the sample received: The results are valid for the sample received.

Additions to, deviations, or exclusions from the test method:

Results on this report belong to the sample(s) defined above only. Analyses indicated with (*) are accredited. This report shall not be partially copied or reproduced without written consent of the laboratory. Testing reports without electronic signature and seal are invalid. No handling is made by our laboratory. In the analysed sample, the technical and legal responsibility for the determination of the procedures and parameters to be checked from the collection of the sample to its delivery to our laboratory belongs to the sampler. Opinions or comments are not made in the test reports.

Seal	Date	Analyst	Approval/Date
TENMAK Bor Araştırma Enstitüsü Dumlupınar Bulvarı (Eskişehir Yolu 7.km.) No:166 D Blok (06560) Çankaya/ANKARA	21.07.2025	Merve ÖZTÜRK	Sezgin ÖZKASAPOĞLU 21.07.2025

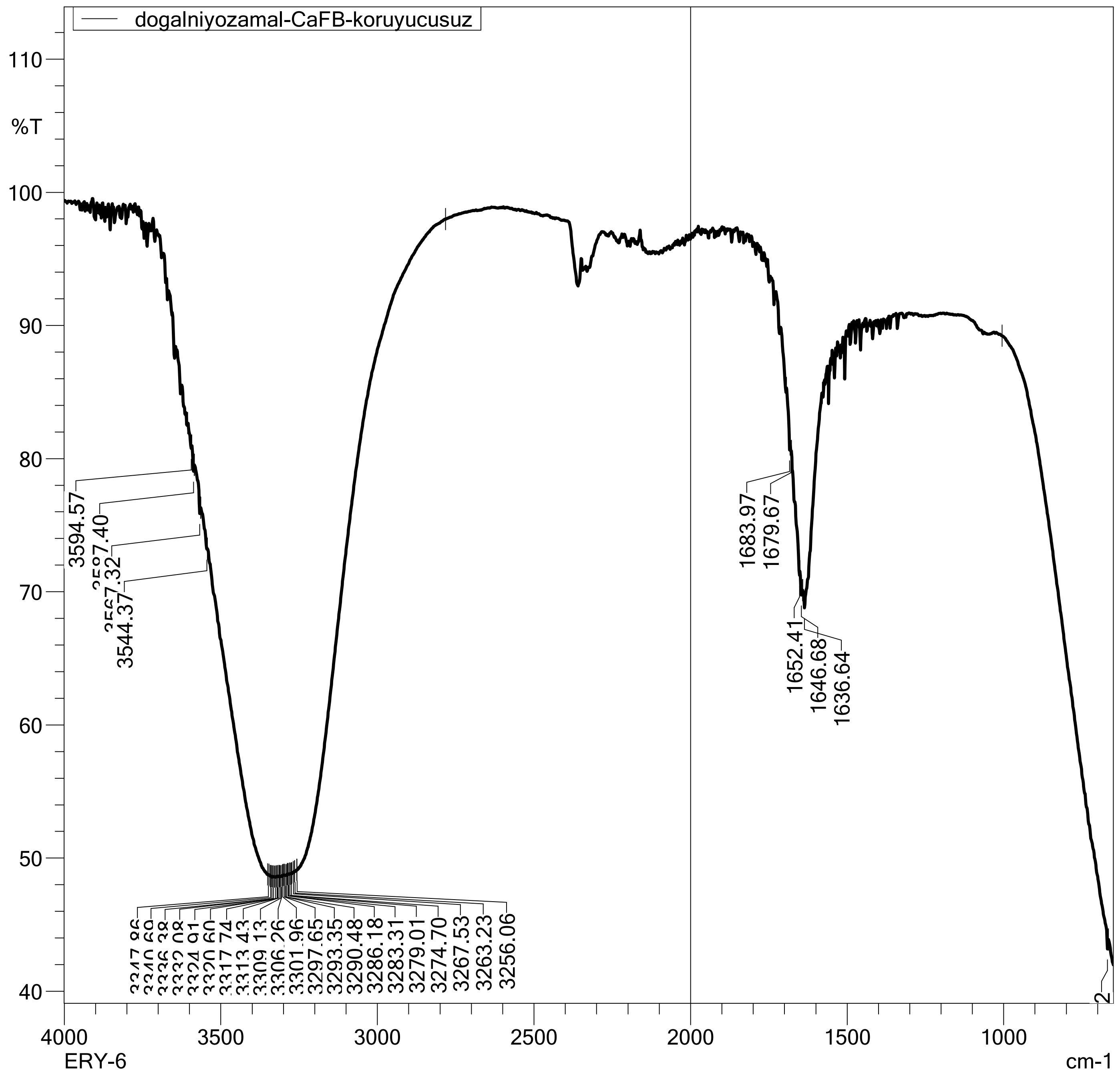

C:¥LabSolutions¥LabSolutionsIR¥Data¥ISMAIL A¥dogalniyozamal-CaFB-koruyucusuz.ispd

ERY-6

	Item	Value
2	Sample name	
3	Sample ID	
4	Option	
5	Intensity Mode	%Transmittance
6	Apodization	Happ-Genzel
9	No. of Scans	25

10	Resolution	4 cm-1

SHIMADZU

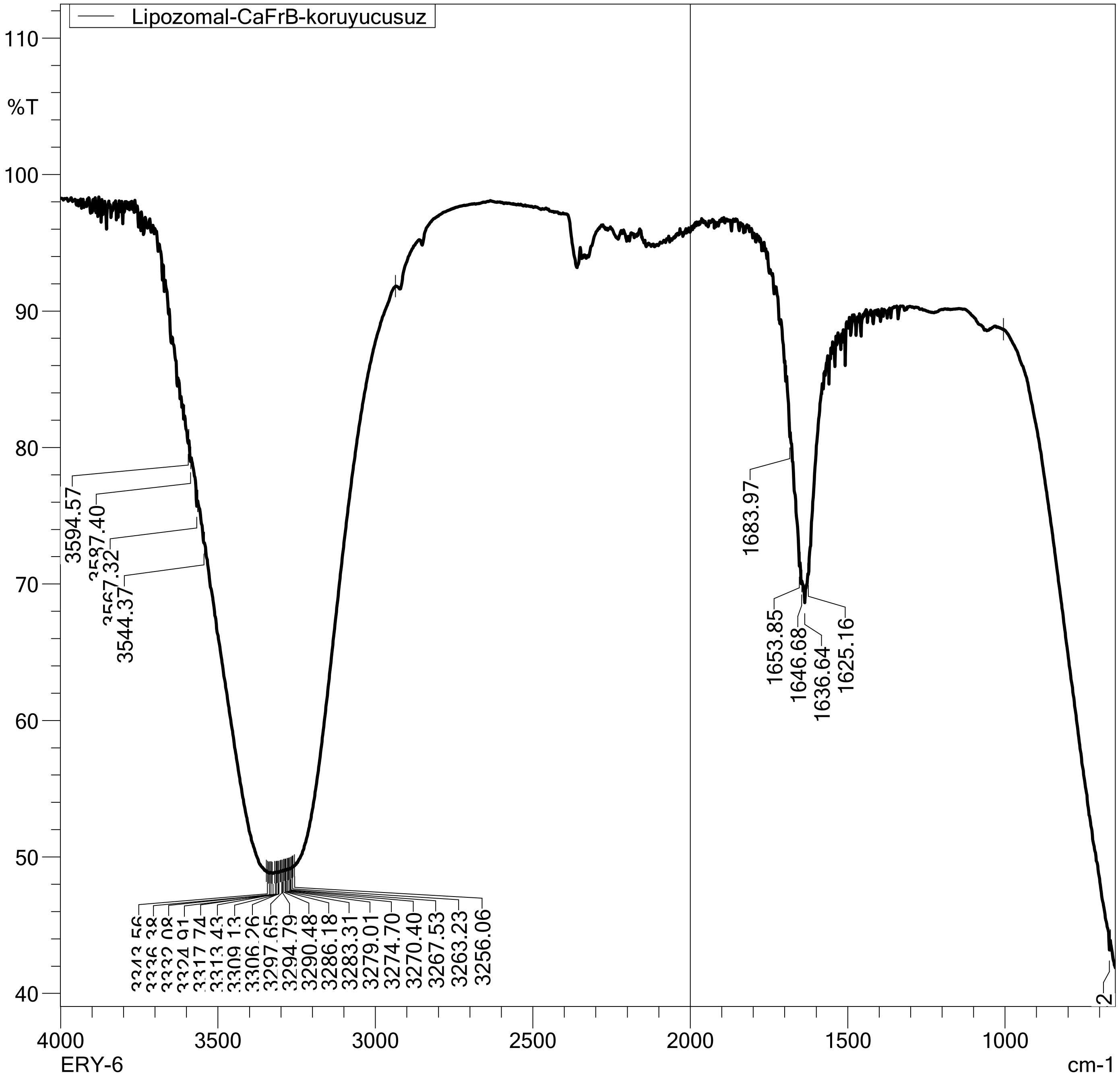

C:¥LabSolutions¥LabSolutionsIR¥Data¥ISMAIL A¥Lipozomal-CaFrB-koruyucusuz.ispd

ERY-6

	Item	Value
2	Sample name	
3	Sample ID	
4	Option	
5	Intensity Mode	%Transmittance
6	Apodization	Happ-Genzel
9	No. of Scans	25

10	Resolution	4 cm-1

SHIMADZU

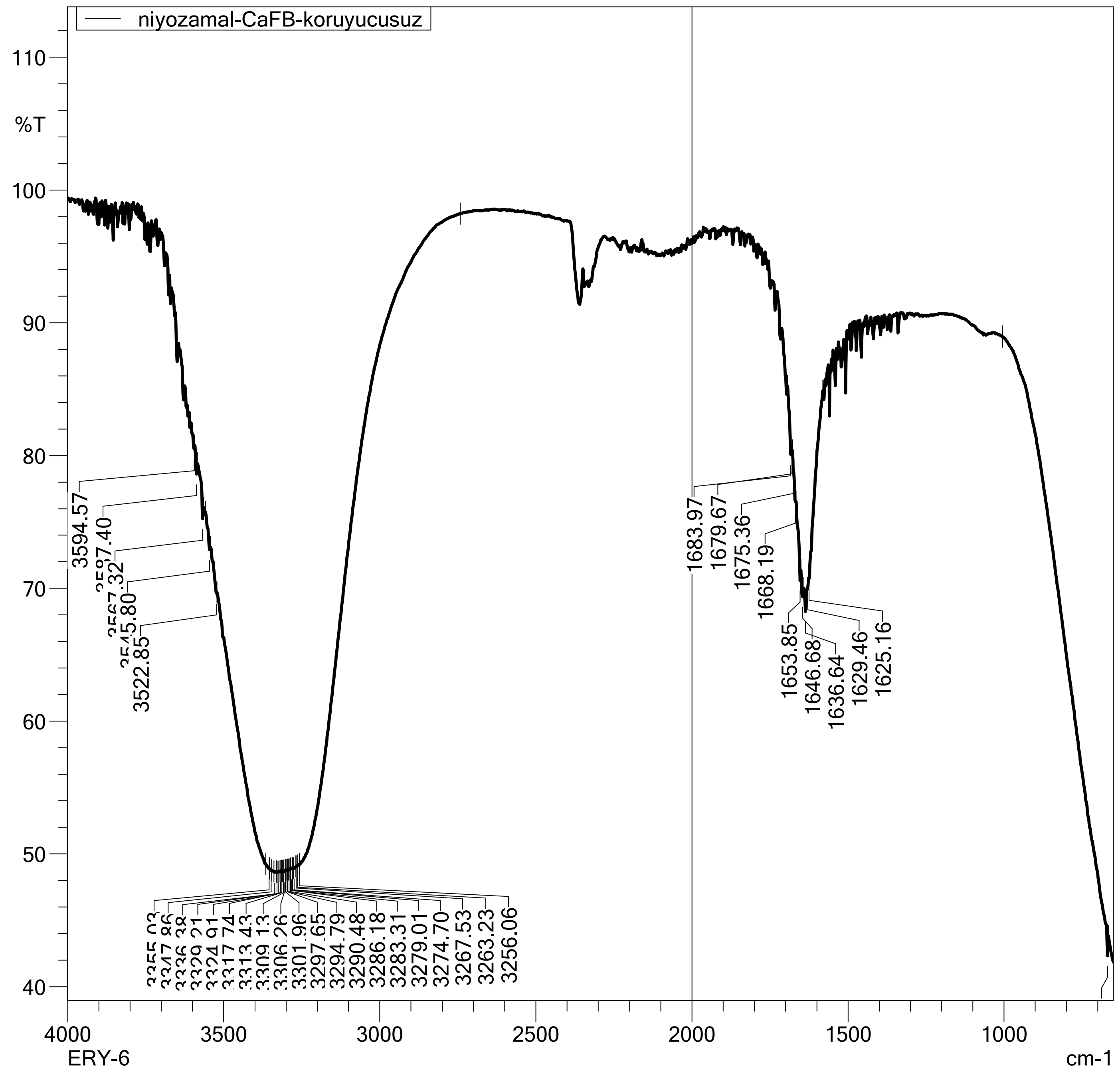

C:¥LabSolutions¥LabSolutionsIR¥Data¥ISMAIL A¥niyozamal-CaFB-koruyucusuz.ispd

ERY-6

	Item	Value
2	Sample name	
3	Sample ID	
4	Option	
5	Intensity Mode	%Transmittance
6	Apodization	Happ-Genzel
9	No. of Scans	25

10	Resolution	4 cm-1

SHIMADZU

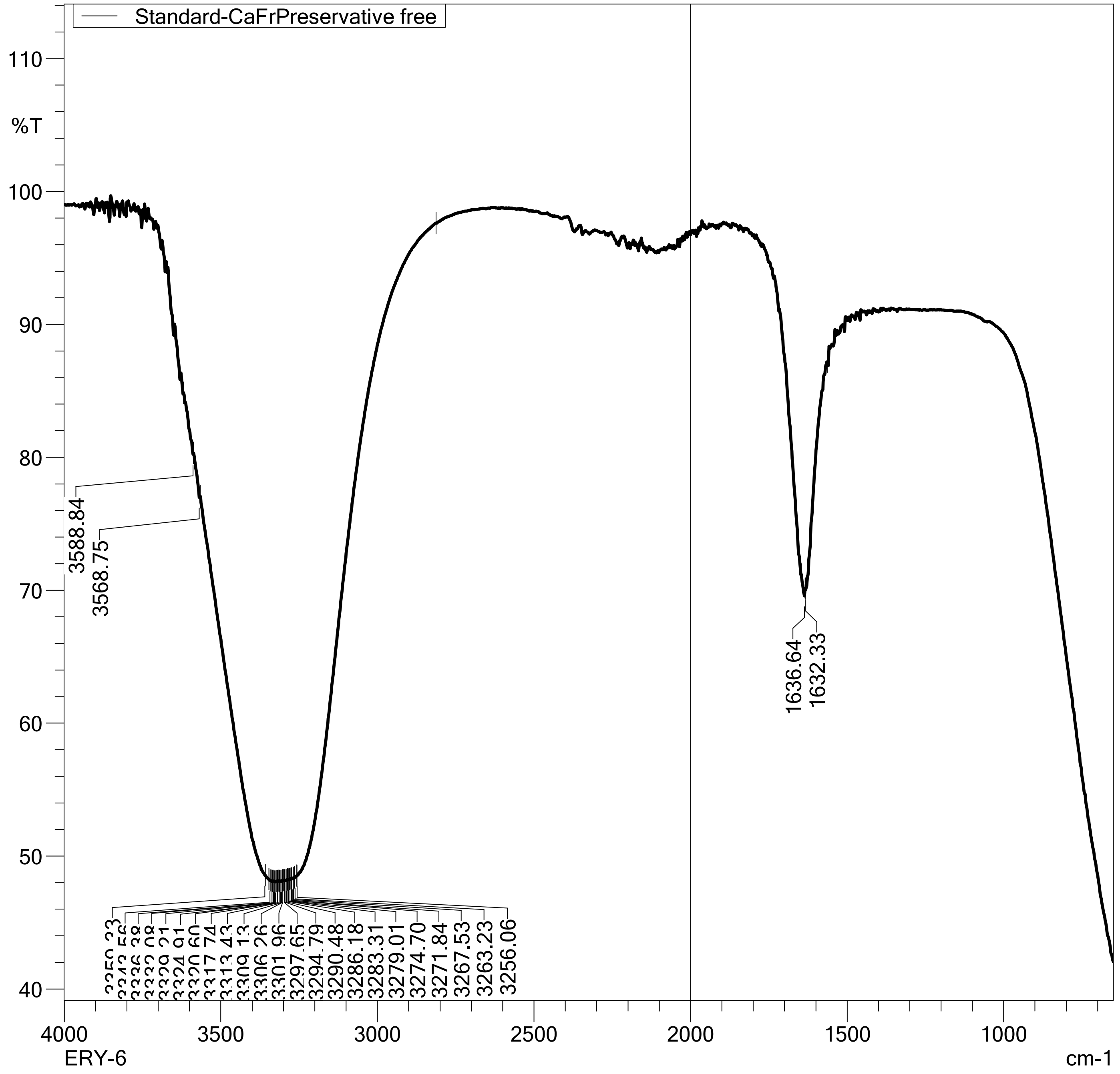

C:¥LabSolutions¥LabSolutionsIR¥Data¥ISMAIL A¥Standard-CaFrPreservative free.ispd

ERY-6

	Item	Value
2	Sample name	
3	Sample ID	
4	Option	
5	Intensity Mode	%Transmittance
6	Apodization	Happ-Genzel
9	No. of Scans	25

10	Resolution	4 cm-1

SFA ARGE
VE ÖZEL SAĞLIK HİZMETLERİ TİC. LTD. ŞTİ.
Sanayi Mah. Teknopark Bulvarı No:1/3a/Z01 Pendik İstanbul
TEL:0216 504 81 82 E-MAIL: info@sfaarge.com

ANALİZ RAPORU/ *ANALYSIS REPORT*

Rapor Numarası / *Report Number* : SFAP250394
Rapor Tarihi / *Report Date* : 11.09.2025
Numuneyi Gönderen Kurum / *Sample Sender Company* : SFA ARGE VE ÖZEL SAĞLIK HİZ. TİC. LTD. ŞTİ. (ARGE)
Talep Teklif Numarası / *Offer Number* :
Numune Kabul Tarihi / *Sample Acceptance Date* : 08.09.2025
Analiz Başlama ve Bitiş Tarihi / *Starting and Ending Date of Analysis* : 08.09.2025 - 08.09.2025
Numune Adı - Türü / *Sample Name - Type* : Doğal niozomal kalsiyum Furoktoborat Koruyucusuz
Ambalaj / *Packing* - **Miktar** / *Amount* : Cam şişe / 50 ml
50 ml
ÜTT-SKT / *PD-ED* - **Seri- LOT** / *Serial number - LOT* : UT:03.09.2025 - SKT:-
Çalışma Şartları(Sıcaklık-Nem) / *Working Conditions (Temp-Humidity)* : Fiziksel Lab. : 25±2°C / %40-70 Bağıl Nem
Numune Saklama Koşulları / *Sample Storage Conditions* : Oda Sıcaklığı

**Analiz** / *Analysis*	**Metot** / *Method*	**Sonuç** / *Result*	**Birim** / *Unit*	**Limit Değer** / *Limit Value*	**Değerlendirme** / *Evaluation*
**Parçacık Boyutu**/*Particle Size*	İşletme İçi Metot FAT 012(REV01) / In-house Method FAT 012(REV01)	152,0	nm	-	-
**Zeta Potansiyel**/*Zeta Potential*	İşletme İçi Metot FAT 012(REV01) / In-house Method FAT 012(REV01)	-29,4	mV	-	-

Fiziksel Analizler Çalışma Detayları /Physical Analysis Study Details

Analizler /*Analysis*	Yöntem / *Method*	Kullanılan Ortam-Cihaz / *Media-Device Used*
**Parçacık Boyutu**/*Particle Size*	Dinamik Işık Saçılımı Tekniği/*Dynamic Light Scattering(DLS)Technique*	Malvern Zetasizer Nano
**Zeta Potansiyel**/*Zeta Potential*	Elektroforetik Işık Saçılımı Tekniği /*Electrophoretic Light Scattering Technique*	Malvern Zetasizer Nano

YORUMLAR/*COMMENTS*
Size-Zeta cihaz çıktıları ekte paylaşılmıştır./*Size-Zeta device outputs are shared in the attachment.*

SFA ARGE
VE ÖZEL SAĞLIK HİZMETLERİ TİC. LTD. ŞTİ.
Sanayi Mah. Teknopark Bulvarı No:1/3a/Z01 Pendik İstanbul
TEL:0216 504 81 82 E-MAIL: info@sfaarge.com

AÇIKLAMALAR / *DESCRIPTIONS*

1. Bu analiz raporu, sadece bu numuneye aittir / *This analysis report belongs only to this sample.*
2. .Bu rapor ve sonuçları SFA ARGE izni olmadan ticari ve reklam amaçlı tamamen veya kısmen çoğaltılamaz veya yayınlanamaz. */This report cannot be copied and duplicated unaware of the SFA R&D.*
3. Analiz yapılan numunede, numunenin alındığından laboratuvarımıza teslimine kadar olan prosedürlerin ve bakılması istenilen grup ve parametrelerin belirlenmesinde teknik ve hukuki sorumluluk numuneyi alana aittir / *In the analyzed sample, the technical and legal responsibility for determining the procedures (from the sample taken to the delivery to our laboratory) and the groups and parameters to be examined belongs to the sender company.*
4. İmzasız analiz sonuç raporları geçersizdir / *Unsigned reports are invalid.*
5. Bu rapor üzerinde revizyon talepleri engeç 30 gün içinde yapılabilir ve revizyonu uygun görüldüğünde gerçekleştirilir / *Revision requests must be made with in 30 days. If the requested revision is appropriate, it is made.*

Ek Açıklama / *Additional Description*

Rabia YAMAN
Numune Kabul Ve Raporlama Birim Sorumlusu / *Sample Acceptance And Reporting Unit Manager*

E-İmzalayan: RABİA YAMAN
Tarih: 11.09.2025

Helya KHOSROPANAH
Fiziksel Analiz Laboratuvarı Birim Sorumlusu / *Physical Analysis Laboratory Unit Manager*

E-İmzalayan: HELYA KHOSROPANAH
Tarih: 11.09.2025

Betül TÜRKER ŞALLI
Laboratuvar Müdürü / *Laboratory Manager*

E-İmzalayan: (Y) RECEP AYDIN
Tarih: 11.09.2025

Size Distribution Report by Intensity

v2.2

Malvern

Sample Details

Sample Name: SFAP250394 1/100 SEY 0.45 MIC 1

SOP Name: mansettings.nano

General Notes:

**File Name:**	Fiziksel Analiz Laboratuv...	**Dispersant Name:**	Water
**Record Number:**	4101	**Dispersant RI:**	1,330
**Material RI:**	1,33	**Viscosity (cP):**	0,8872
**Material Absorbtion:**	0,100	**Measurement Date and Time:**	8 Eylül 2025 Pazartesi 16:5...

System

**Temperature (°C):**	25,0	**Duration Used (s):**	60
**Count Rate (kcps):**	303,1	**Measurement Position (mm):**	5,50
**Cell Description:**	Clear disposable zeta cell	**Attenuator:**	6

Results

			**Size (d.n...**	**% Intensity:**	**St Dev (d.n...**
**Z-Average (d.nm):**	152,0	**Peak 1:**	174,1	98,8	71,82
**PdI:**	0,168	**Peak 2:**	4733	1,2	759,3
**Intercept:**	0,958	**Peak 3:**	0,000	0,0	0,000

Result quality **Good**

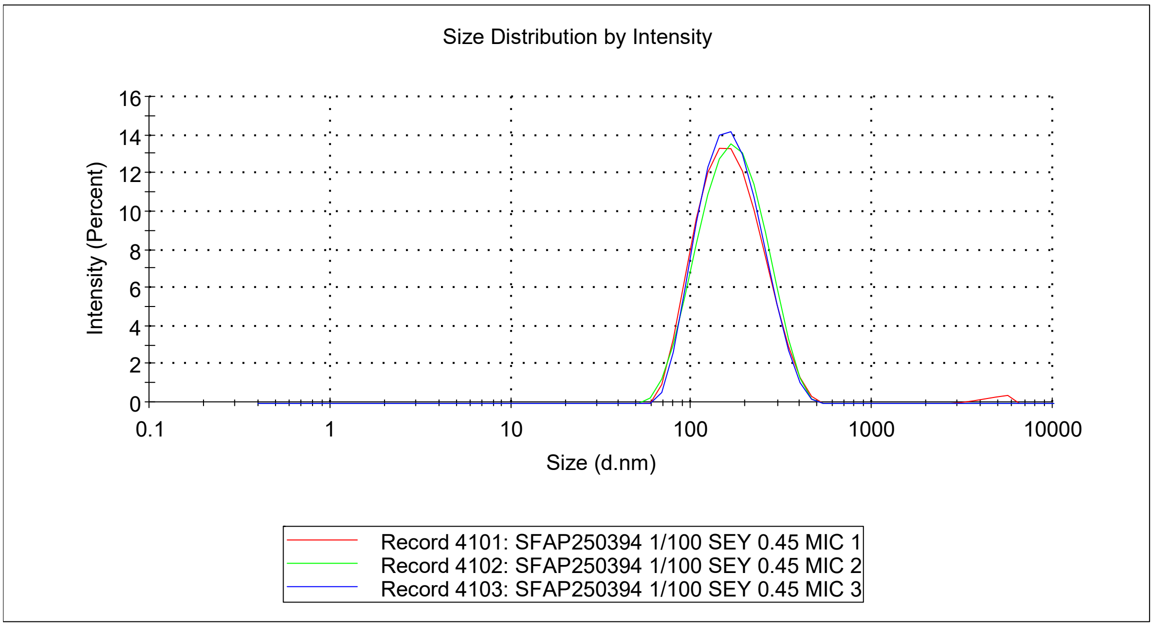

Zeta Potential Report

v2.3

Malvern

Malvern Instruments Ltd - © Copyright 2008

Sample Details

Sample Name: SFAP250394 1/100 SEY 0.45 MIC 1

SOP Name: mansettings.nano

General Notes:

File Name: Fiziksel Analiz Laboratuvarı....

Record Number: 4104

Date and Time: 8 Eylül 2025 Pazartesi 17:04:08

Dispersant Name: Water

Dispersant RI: 1,330

Viscosity (cP): 0,8872

Dispersant Dielectric Constant: 78,5

System

Temperature (°C): 25,0

Count Rate (kcps): 136,5

Cell Description: Clear disposable zeta c...

Zeta Runs: 12

Measurement Position (mm): 2,00

Attenuator: 6

Results

			Mean (mV)	Area (%)	St Dev (mV)
**Zeta Potential (mV):**	-29,4	**Peak 1:**	-29,4	100,0	5,82
**Zeta Deviation (mV):**	5,82	**Peak 2:**	0,00	0,0	0,00
**Conductivity (mS/cm):**	0,0296	**Peak 3:**	0,00	0,0	0,00
**Result quality**	Good				

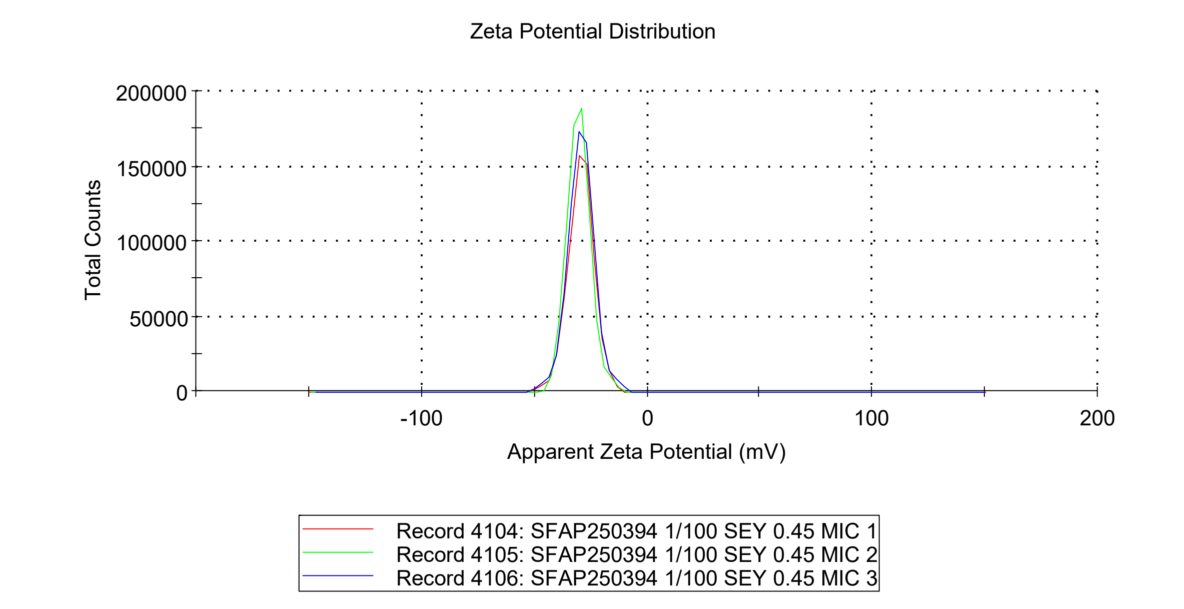

SFA ARGE
VE ÖZEL SAĞLIK HİZMETLERİ TİC. LTD. ŞTİ.
Sanayi Mah. Teknopark Bulvarı No:1/3a/Z01 Pendik İstanbul
TEL:0216 504 81 82 E-MAIL: info@sfaarge.com

ANALİZ RAPORU/ *ANALYSIS REPORT*

Rapor Numarası / *Report Number* : SFAP250395
Rapor Tarihi / *Report Date* : 11.09.2025
Numuneyi Gönderen Kurum / *Sample Sender Company* : SFA ARGE VE ÖZEL SAĞLIK HİZ. TİC. LTD. ŞTİ. (ARGE)
Talep Teklif Numarası / *Offer Number* :
Numune Kabul Tarihi / *Sample Acceptance Date* : 08.09.2025
Analiz Başlama ve Bitiş Tarihi / *Starting and Ending Date of Analysis* : 08.09.2025 - 08.09.2025
Numune Adı - Türü / *Sample Name - Type* : Lipozomal kalsiyum Furoktoborat Koruyucusuz
Ambalaj / *Packing* - **Miktar** / *Amount* : Cam şişe / 50 ml
50 ml
ÜTT-SKT / *PD-ED* - **Seri- LOT** / *Serial number - LOT* : UT:03.09.2025 - SKT:-
Çalışma Şartları(Sıcaklık-Nem) / *Working Conditions (Temp-Humidity)* : Fiziksel Lab. : 25±2°C / %40-70 Bağıl Nem
Numune Saklama Koşulları / *Sample Storage Conditions* : Oda Sıcaklığı

**Analiz** / *Analysis*	**Metot** / *Method*	**Sonuç** / *Result*	**Birim** / *Unit*	**Limit Değer** / *Limit Value*	**Değerlendirme** / *Evaluation*
**Parçacık Boyutu**/*Particle Size*	İşletme İçi Metot FAT 012(REV01) / In-house Method FAT 012(REV01)	88,29	nm	-	-
**Zeta Potansiyel**/*Zeta Potential*	İşletme İçi Metot FAT 012(REV01) / In-house Method FAT 012(REV01)	-25,1	mV	-	-

Fiziksel Analizler Çalışma Detayları /Physical Analysis Study Details		
Analizler /*Analysis*	Yöntem / *Method*	Kullanılan Ortam-Cihaz / *Media-Device Used*
**Parçacık Boyutu**/*Particle Size*	Dinamik Işık Saçılımı Tekniği/*Dynamic Light Scattering(DLS)Technique*	Malvern Zetasizer Nano
**Zeta Potansiyel**/*Zeta Potential*	Elektroforetik Işık Saçılımı Tekniği /*Electrophoretic Light Scattering Technique*	Malvern Zetasizer Nano

YORUMLAR/*COMMENTS*
Size-Zeta cihaz çıktıları ekte paylaşılmıştır./*Size-Zeta device outputs are shared in the attachment.*

SFA ARGE
VE ÖZEL SAĞLIK HİZMETLERİ TİC. LTD. ŞTİ.
Sanayi Mah. Teknopark Bulvarı No:1/3a/Z01 Pendik İstanbul
TEL:0216 504 81 82 E-MAIL: info@sfaarge.com

AÇIKLAMALAR / *DESCRIPTIONS*

1. Bu analiz raporu, sadece bu numuneye aittir / *This analysis report belongs only to this sample.*
2. .Bu rapor ve sonuçları SFA ARGE izni olmadan ticari ve reklam amaçlı tamamen veya kısmen çoğaltılamaz veya yayınlanamaz. */This report cannot be copied and duplicated unaware of the SFA R&D.*
3. Analiz yapılan numunede, numunenin alındığından laboratuvarımıza teslimine kadar olan prosedürlerin ve bakılması istenilen grup ve parametrelerin belirlenmesinde teknik ve hukuki sorumluluk numuneyi alana aittir / *In the analyzed sample, the technical and legal responsibility for determining the procedures (from the sample taken to the delivery to our laboratory) and the groups and parameters to be examined belongs to the sender company.*
4. İmzasız analiz sonuç raporları geçersizdir / *Unsigned reports are invalid.*
5. Bu rapor üzerinde revizyon talepleri engeç 30 gün içinde yapılabilir ve revizyonu uygun görüldüğünde gerçekleştirilir / *Revision requests must be made with in 30 days. If the requested revision is appropriate, it is made.*

Ek Açıklama / *Additional Description*

Rabia YAMAN
Numune Kabul Ve Raporlama Birim Sorumlusu / *Sample Acceptance And Reporting Unit Manager*

E-İmzalayan: RABİA YAMAN
Tarih: 11.09.2025

Helya KHOSROPANAH
Fiziksel Analiz Laboratuvarı Birim Sorumlusu / *Physical Analysis Laboratory Unit Manager*

E-İmzalayan: HELYA KHOSROPANAH
Tarih: 11.09.2025

Betül TÜRKER ŞALLI
Laboratuvar Müdürü / *Laboratory Manager*

E-İmzalayan: (Y) RECEP AYDIN
Tarih: 11.09.2025

Size Distribution Report by Intensity

v2.2

Malvern

Sample Details

Sample Name: SFAP250395 1/100 SEY 0.45 MIC 1

SOP Name: mansettings.nano

General Notes:

**File Name:**	Fiziksel Analiz Laboratuv...	**Dispersant Name:**	Water
**Record Number:**	4083	**Dispersant RI:**	1,330
**Material RI:**	1,33	**Viscosity (cP):**	0,8872
**Material Absorbtion:**	0,100	**Measurement Date and Time:**	8 Eylül 2025 Pazartesi 16:0...

System

**Temperature (°C):**	25,0	**Duration Used (s):**	60
**Count Rate (kcps):**	288,3	**Measurement Position (mm):**	5,50
**Cell Description:**	Clear disposable zeta cell	**Attenuator:**	7

Results

			**Size (d.n...**	**% Intensity:**	**St Dev (d.n...**
**Z-Average (d.nm):**	88,29	**Peak 1:**	106,1	95,7	57,30
**PdI:**	0,248	**Peak 2:**	3906	4,3	1115
**Intercept:**	0,948	**Peak 3:**	0,000	0,0	0,000

Result quality **Good**

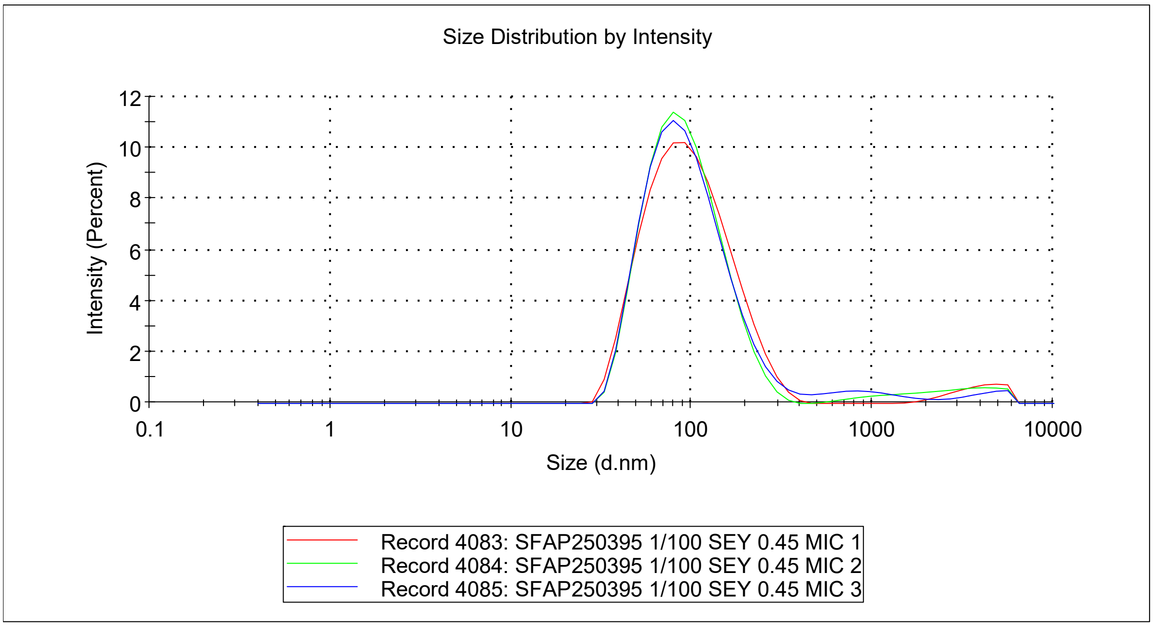

Zeta Potential Report

v2.3

Malvern Instruments Ltd - © Copyright 2008

Sample Details

Sample Name: SFAP250395 1/100 SEY 0.45 MIC 1

SOP Name: mansettings.nano

General Notes:

File Name: Fiziksel Analiz Laboratuvarı....
Dispersant Name: Water

Record Number: 4086
Dispersant RI: 1,330

Date and Time: 8 Eylül 2025 Pazartesi 16:07:48
Viscosity (cP): 0,8872

Dispersant Dielectric Constant: 78,5

System

Temperature (°C): 25,0
Zeta Runs: 12

Count Rate (kcps): 76,7
Measurement Position (mm): 2,00

Cell Description: Clear disposable zeta c...
Attenuator: 8

Results

			Mean (mV)	Area (%)	St Dev (mV)
**Zeta Potential (mV):**	-25,1	**Peak 1:**	-25,1	100,0	6,60
**Zeta Deviation (mV):**	6,60	**Peak 2:**	0,00	0,0	0,00
**Conductivity (mS/cm):**	0,0209	**Peak 3:**	0,00	0,0	0,00
**Result quality**	Good				

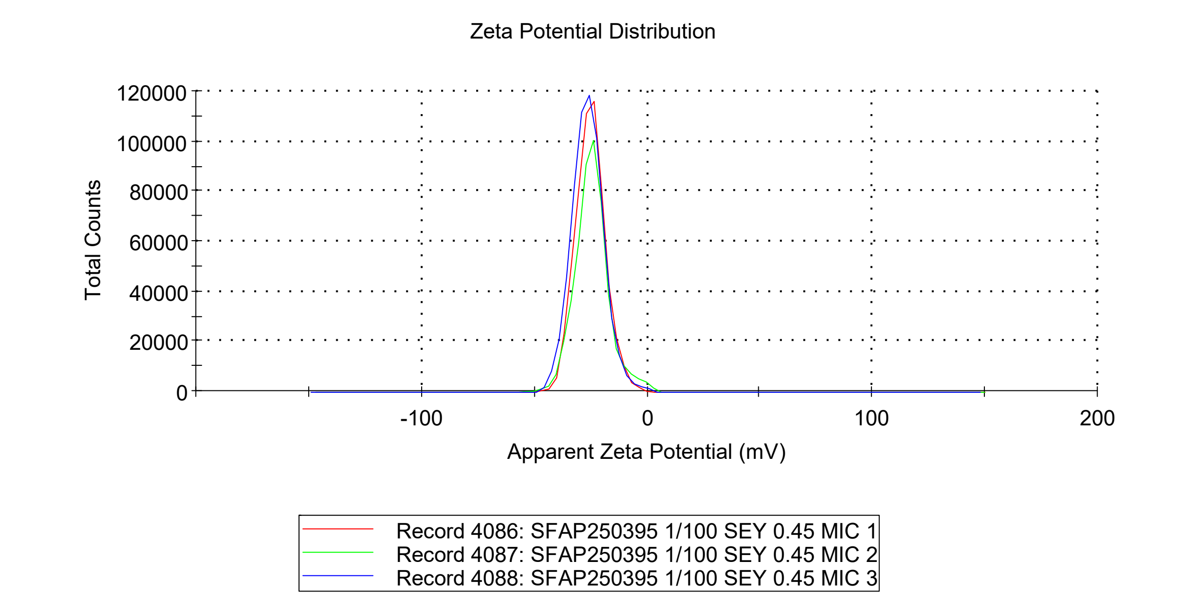

SFA ARGE
VE ÖZEL SAĞLIK HİZMETLERİ TİC. LTD. ŞTİ.
Sanayi Mah. Teknopark Bulvarı No:1/3a/Z01 Pendik İstanbul
TEL:0216 504 81 82 E-MAIL: info@sfaarge.com

ANALİZ RAPORU/ *ANALYSIS REPORT*

**Rapor Numarası** / *Report Number* :	SFAP250397
**Rapor Tarihi** / *Report Date* :	11.09.2025
**Numuneyi Gönderen Kurum** / *Sample Sender Company* :	SFA ARGE VE ÖZEL SAĞLIK HİZ. TİC. LTD. ŞTİ. (ARGE)
**Talep Teklif Numarası** / *Offer Number* :	
**Numune Kabul Tarihi** / *Sample Acceptance Date* :	08.09.2025
**Analiz Başlama ve Bitiş Tarihi** / *Starting and Ending Date of Analysis* :	08.09.2025 - 08.09.2025
**Numune Adı - Türü** / *Sample Name - Type* :	Niozomal kalsiyum Furoktoborat Koruyucusuz
**Ambalaj** / *Packing* - **Miktar** / *Amount* :	50ML/CAM AMBALAJ<br>50ML
**ÜTT-SKT** / *PD-ED* - **Seri- LOT** / *Serial number - LOT* :	UT:03.09.2025 - SKT:-
**Çalışma Şartları(Sıcaklık-Nem)** / *Working Conditions (Temp-Humidity)* :	Fiziksel Lab. : 25±2°C / %40-70 Bağıl Nem
**Numune Saklama Koşulları** / *Sample Storage Conditions* :	Oda Sıcaklığı

**Analiz** / *Analysis*	**Metot** / *Method*	**Sonuç** / *Result*	**Birim** / *Unit*	**Limit Değer** / *Limit Value*	**Değerlendirme** / *Evaluation*
**Parçacık Boyutu**/*Particle Size*	İşletme İçi Metot FAT 012(REV01) / In-house Method FAT 012(REV01)	136,8	nm	-	-
**Zeta Potansiyel**/*Zeta Potential*	İşletme İçi Metot FAT 012(REV01) / In-house Method FAT 012(REV01)	-26,7	mV	-	-

Fiziksel Analizler Çalışma Detayları /Physical Analysis Study Details		
Analizler /*Analysis*	Yöntem / *Method*	Kullanılan Ortam-Cihaz / *Media-Device Used*
**Parçacık Boyutu**/*Particle Size*	Dinamik Işık Saçılımı Tekniği/*Dynamic Light Scattering(DLS)Technique*	Malvern Zetasizer Nano
**Zeta Potansiyel**/*Zeta Potential*	Elektroforetik Işık Saçılımı Tekniği /*Electrophoretic Light Scattering Technique*	Malvern Zetasizer Nano

YORUMLAR/*COMMENTS*

Size-Zeta cihaz çıktıları ekte paylaşılmıştır./*Size-Zeta device outputs are shared in the attachment.*

SFA ARGE
VE ÖZEL SAĞLIK HİZMETLERİ TİC. LTD. ŞTİ.
Sanayi Mah. Teknopark Bulvarı No:1/3a/Z01 Pendik İstanbul
TEL:0216 504 81 82 E-MAIL: info@sfaarge.com

AÇIKLAMALAR / *DESCRIPTIONS*

1. Bu analiz raporu, sadece bu numuneye aittir / *This analysis report belongs only to this sample.*
2. .Bu rapor ve sonuçları SFA ARGE izni olmadan ticari ve reklam amaçlı tamamen veya kısmen çoğaltılamaz veya yayınlanamaz. */This report cannot be copied and duplicated unaware of the SFA R&D.*
3. Analiz yapılan numunede, numunenin alındığından laboratuvarımıza teslimine kadar olan prosedürlerin ve bakılması istenilen grup ve parametrelerin belirlenmesinde teknik ve hukuki sorumluluk numuneyi alana aittir / *In the analyzed sample, the technical and legal responsibility for determining the procedures (from the sample taken to the delivery to our laboratory) and the groups and parameters to be examined belongs to the sender company.*
4. İmzasız analiz sonuç raporları geçersizdir / *Unsigned reports are invalid.*
5. Bu rapor üzerinde revizyon talepleri engeç 30 gün içinde yapılabilir ve revizyonu uygun görüldüğünde gerçekleştirilir / *Revision requests must be made with in 30 days. If the requested revision is appropriate, it is made.*

Ek Açıklama / *Additional Description*

Rabia YAMAN
Numune Kabul Ve Raporlama Birim Sorumlusu / *Sample Acceptance And Reporting Unit Manager*

E-İmzalayan: RABİA YAMAN
Tarih: 11.09.2025

Helya KHOSROPANAH
Fiziksel Analiz Laboratuvarı Birim Sorumlusu / *Physical Analysis Laboratory Unit Manager*

E-İmzalayan: HELYA KHOSROPANAH
Tarih: 11.09.2025

Betül TÜRKER ŞALLI
Laboratuvar Müdürü / *Laboratory Manager*

E-İmzalayan: (Y) RECEP AYDIN
Tarih: 11.09.2025

Size Distribution Report by Intensity

v2.2

Malvern

Sample Details

Sample Name: SFAP250397 1/100 SEY 0.45 MIC 1

SOP Name: mansettings.nano

General Notes:

**File Name:**	Fiziksel Analiz Laboratuv...	**Dispersant Name:**	Water
**Record Number:**	4113	**Dispersant RI:**	1,330
**Material RI:**	1,33	**Viscosity (cP):**	0,8872
**Material Absorbtion:**	0,100	**Measurement Date and Time:**	8 Eylül 2025 Pazartesi 17:3...

System

**Temperature (°C):**	25,0	**Duration Used (s):**	70
**Count Rate (kcps):**	215,5	**Measurement Position (mm):**	5,50
**Cell Description:**	Clear disposable zeta cell	**Attenuator:**	7

Results

			**Size (d.n...**	**% Intensity:**	**St Dev (d.n...**
**Z-Average (d.nm):**	136,8	**Peak 1:**	167,5	100,0	79,55
**PdI:**	0,175	**Peak 2:**	0,000	0,0	0,000
**Intercept:**	0,954	**Peak 3:**	0,000	0,0	0,000

Result quality Good

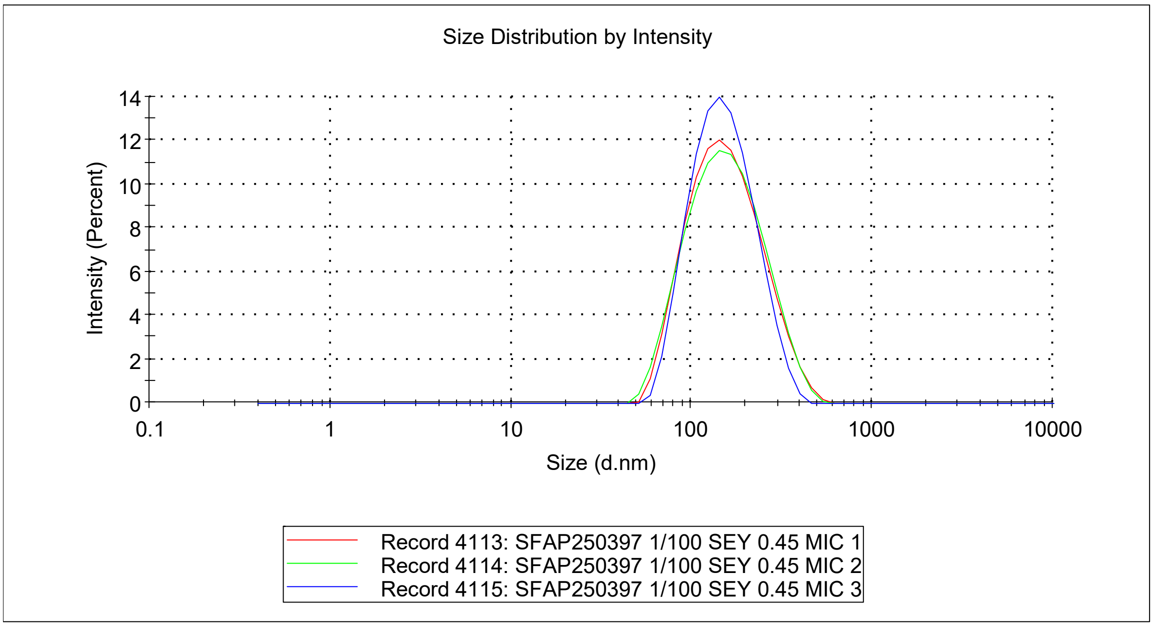

Zeta Potential Report

v2.3

Malvern Instruments Ltd - © Copyright 2008

Sample Details

Sample Name: SFAP250397 1/100 SEY 0.45 MIC 1

SOP Name: mansettings.nano

General Notes:

**File Name:**	Fiziksel Analiz Laboratuvarı....	**Dispersant Name:**	Water
**Record Number:**	4116	**Dispersant RI:**	1,330
**Date and Time:**	8 Eylül 2025 Pazartesi 17:36:55	**Viscosity (cP):**	0,8872
		**Dispersant Dielectric Constant:**	78,5

System

**Temperature (°C):**	25,0	**Zeta Runs:**	12
**Count Rate (kcps):**	180,6	**Measurement Position (mm):**	2,00
**Cell Description:**	Clear disposable zeta c...	**Attenuator:**	7

Results

			Mean (mV)	Area (%)	St Dev (mV)
**Zeta Potential (mV):**	-26,7	**Peak 1:**	-26,7	100,0	6,31
**Zeta Deviation (mV):**	6,31	**Peak 2:**	0,00	0,0	0,00
**Conductivity (mS/cm):**	0,0232	**Peak 3:**	0,00	0,0	0,00
**Result quality**	Good				

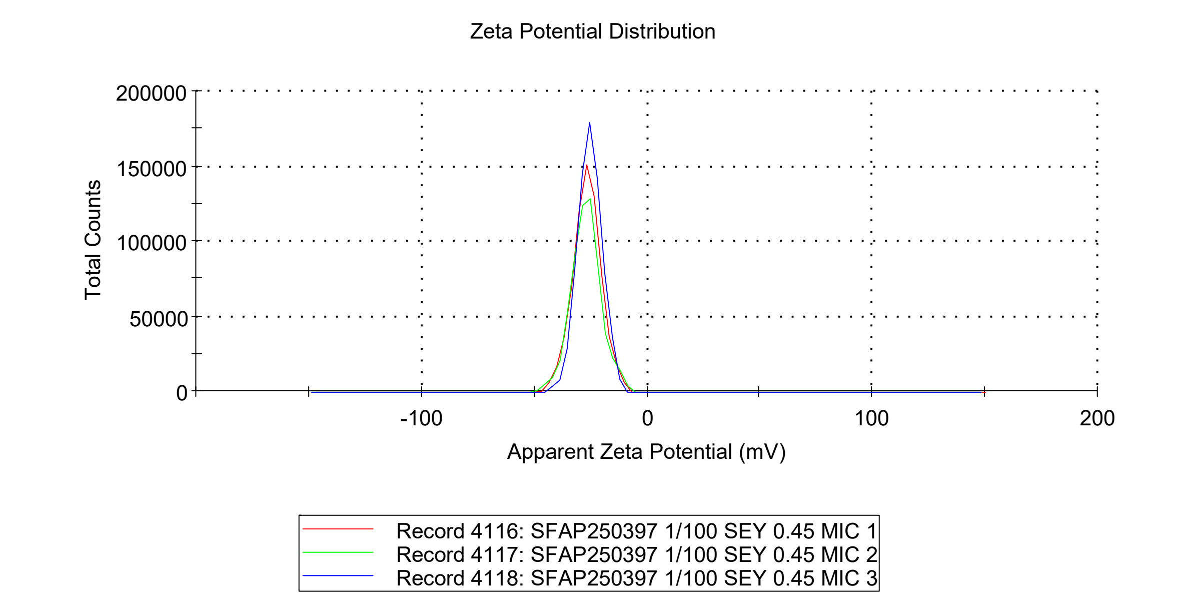

SFA ARGE
VE ÖZEL SAĞLIK HİZMETLERİ TİC. LTD. ŞTİ.
Sanayi Mah. Teknopark Bulvarı No:1/3a/Z01 Pendik İstanbul
TEL:0216 504 81 82 E-MAIL: info@sfaarge.com

ANALİZ RAPORU/ *ANALYSIS REPORT*

**Rapor Numarası** / *Report Number* :	SFAP250399
**Rapor Tarihi** / *Report Date* :	11.09.2025
**Numuneyi Gönderen Kurum** / *Sample Sender Company* :	SFA ARGE VE ÖZEL SAĞLIK HİZ. TİC. LTD. ŞTİ. (ARGE)
**Talep Teklif Numarası** / *Offer Number* :	
**Numune Kabul Tarihi** / *Sample Acceptance Date* :	08.09.2025
**Analiz Başlama ve Bitiş Tarihi** / *Starting and Ending Date of Analysis* :	08.09.2025 - 08.09.2025
**Numune Adı - Türü** / *Sample Name - Type* :	Standart kalsiyum Furoktoborat Koruyucusuz
**Ambalaj** / *Packing* - **Miktar** / *Amount* :	50ML/ CAM AMBALAJ<br>50ML
**ÜTT-SKT** / *PD-ED* - **Seri- LOT** / *Serial number - LOT* :	UT:03.09.2025 - SKT:-
**Çalışma Şartları(Sıcaklık-Nem)** / *Working Conditions (Temp-Humidity)* :	Fiziksel Lab. : 25±2°C / %40-70 Bağıl Nem
**Numune Saklama Koşulları** / *Sample Storage Conditions* :	Oda Sıcaklığı

**Analiz** / *Analysis*	**Metot** / *Method*	**Sonuç** / *Result*	**Birim** / *Unit*	**Limit Değer** / *Limit Value*	**Değerlendirme** / *Evaluation*
**Parçacık Boyutu**/*Particle Size*	İşletme İçi Metot FAT 012(REV01) / In-house Method FAT 012(REV01)	111,7	nm	-	-
**Zeta Potansiyel**/*Zeta Potential*	İşletme İçi Metot FAT 012(REV01) / In-house Method FAT 012(REV01)	-10,0	mV	-	-

Fiziksel Analizler Çalışma Detayları /Physical Analysis Study Details		
Analizler /*Analysis*	Yöntem / *Method*	Kullanılan Ortam-Cihaz / *Media-Device Used*
**Parçacık Boyutu**/*Particle Size*	Dinamik Işık Saçılımı Tekniği/*Dynamic Light Scattering(DLS)Technique*	Malvern Zetasizer Nano
**Zeta Potansiyel**/*Zeta Potential*	Elektroforetik Işık Saçılımı Tekniği /*Electrophoretic Light Scattering Technique*	Malvern Zetasizer Nano

YORUMLAR/*COMMENTS*

Size-Zeta cihaz çıktıları ekte paylaşılmıştır./*Size-Zeta device outputs are shared in the attachment.*

Doküman No:FR 78 006 01
İlk Yayın Tarihi:10.09.2021

Revizyon No 00
Revizyon Tarihi 00

SFA ARGE
VE ÖZEL SAĞLIK HİZMETLERİ TİC. LTD. ŞTİ.
Sanayi Mah. Teknopark Bulvarı No:1/3a/Z01 Pendik İstanbul
TEL:0216 504 81 82 E-MAIL: info@sfaarge.com

AÇIKLAMALAR / *DESCRIPTIONS*

1. Bu analiz raporu, sadece bu numuneye aittir / *This analysis report belongs only to this sample.*
2. .Bu rapor ve sonuçları SFA ARGE izni olmadan ticari ve reklam amaçlı tamamen veya kısmen çoğaltılamaz veya yayınlanamaz. */This report cannot be copied and duplicated unaware of the SFA R&D.*
3. Analiz yapılan numunede, numunenin alındığından laboratuvarımıza teslimine kadar olan prosedürlerin ve bakılması istenilen grup ve parametrelerin belirlenmesinde teknik ve hukuki sorumluluk numuneyi alana aittir / *In the analyzed sample, the technical and legal responsibility for determining the procedures (from the sample taken to the delivery to our laboratory) and the groups and parameters to be examined belongs to the sender company.*
4. İmzasız analiz sonuç raporları geçersizdir / *Unsigned reports are invalid.*
5. Bu rapor üzerinde revizyon talepleri engeç 30 gün içinde yapılabilir ve revizyonu uygun görüldüğünde gerçekleştirilir / *Revision requests must be made with in 30 days. If the requested revision is appropriate, it is made.*

Ek Açıklama / *Additional Description*

Rabia YAMAN
Numune Kabul Ve Raporlama Birim Sorumlusu / *Sample Acceptance And Reporting Unit Manager*

E-İmzalayan: RABİA YAMAN
Tarih: 11.09.2025

Helya KHOSROPANAH
Fiziksel Analiz Laboratuvarı Birim Sorumlusu / *Physical Analysis Laboratory Unit Manager*

E-İmzalayan: HELYA KHOSROPANAH
Tarih: 11.09.2025

Betül TÜRKER ŞALLI
Laboratuvar Müdürü / *Laboratory Manager*

E-İmzalayan: (Y) RECEP AYDIN
Tarih: 11.09.2025

Size Distribution Report by Intensity

v2.2

Malvern

Sample Details

**Sample Name:**	SFAP250399 1/100 SEY 0.45 MIC 1
**SOP Name:**	mansettings.nano
**General Notes:**	

**File Name:**	Fiziksel Analiz Laboratuv...	**Dispersant Name:**	Water
**Record Number:**	4068	**Dispersant RI:**	1,330
**Material RI:**	1,59	**Viscosity (cP):**	0,8872
**Material Absorbtion:**	0,010	**Measurement Date and Time:**	8 Eylül 2025 Pazartesi 15:2...

System

**Temperature (°C):**	25,0	**Duration Used (s):**	60
**Count Rate (kcps):**	443,6	**Measurement Position (mm):**	5,50
**Cell Description:**	Clear disposable zeta cell	**Attenuator:**	10

Results

			Size (d.n...	% Intensity:	St Dev (d.n...
**Z-Average (d.nm):**	111,7	**Peak 1:**	229,2	89,2	152,9
**PdI:**	0,602	**Peak 2:**	2,113	10,8	0,5792
**Intercept:**	0,126	**Peak 3:**	0,000	0,0	0,000

Result quality **Good**

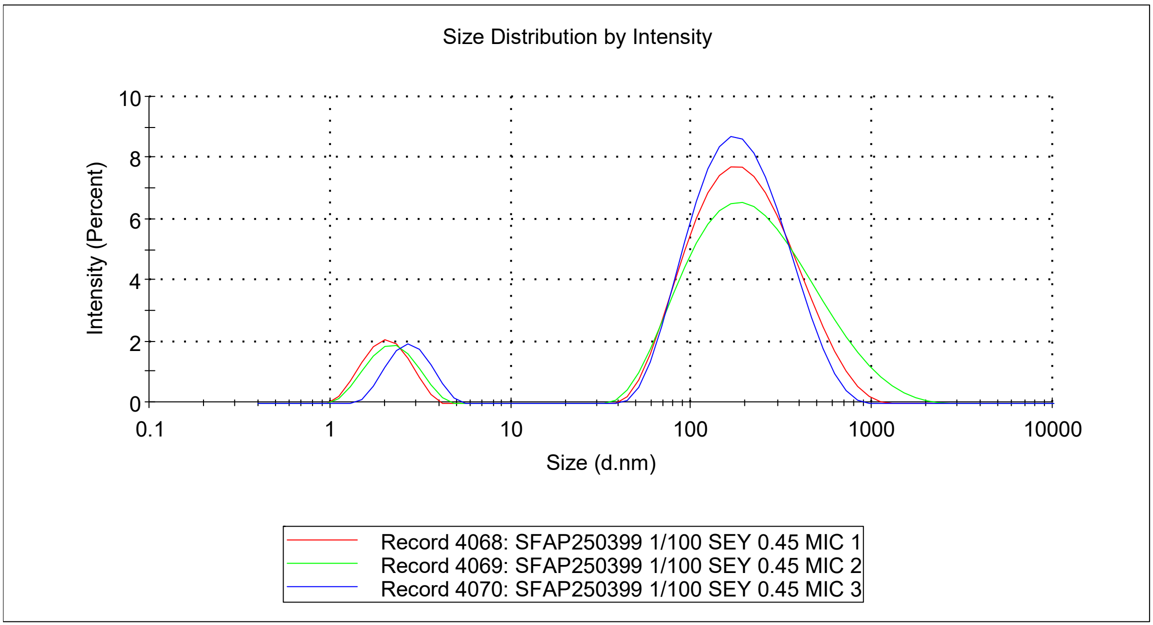

Zeta Potential Report
v2.3

Malvern

Malvern Instruments Ltd - © Copyright 2008

Sample Details

**Sample Name:**	SFAP250399 1/100 SEY 0.45 MIC 1
**SOP Name:**	mansettings.nano
**General Notes:**	

**File Name:**	Fiziksel Analiz Laboratuvarı....	**Dispersant Name:**	Water
**Record Number:**	4071	**Dispersant RI:**	1,330
**Date and Time:**	8 Eylül 2025 Pazartesi 15:25:35	**Viscosity (cP):**	0,8872
		**Dispersant Dielectric Constant:**	78,5

System

**Temperature (°C):**	25,0	**Zeta Runs:**	43
**Count Rate (kcps):**	36,4	**Measurement Position (mm):**	2,00
**Cell Description:**	Clear disposable zeta c...	**Attenuator:**	11

Results

			Mean (mV)	Area (%)	St Dev (mV)
**Zeta Potential (mV):**	-10,0	**Peak 1:**	-2,64	81,3	13,3
**Zeta Deviation (mV):**	72,5	**Peak 2:**	119	10,3	2,42
**Conductivity (mS/cm):**	0,0157	**Peak 3:**	40,6	2,8	6,43
**Result quality**	**See result quality report**				

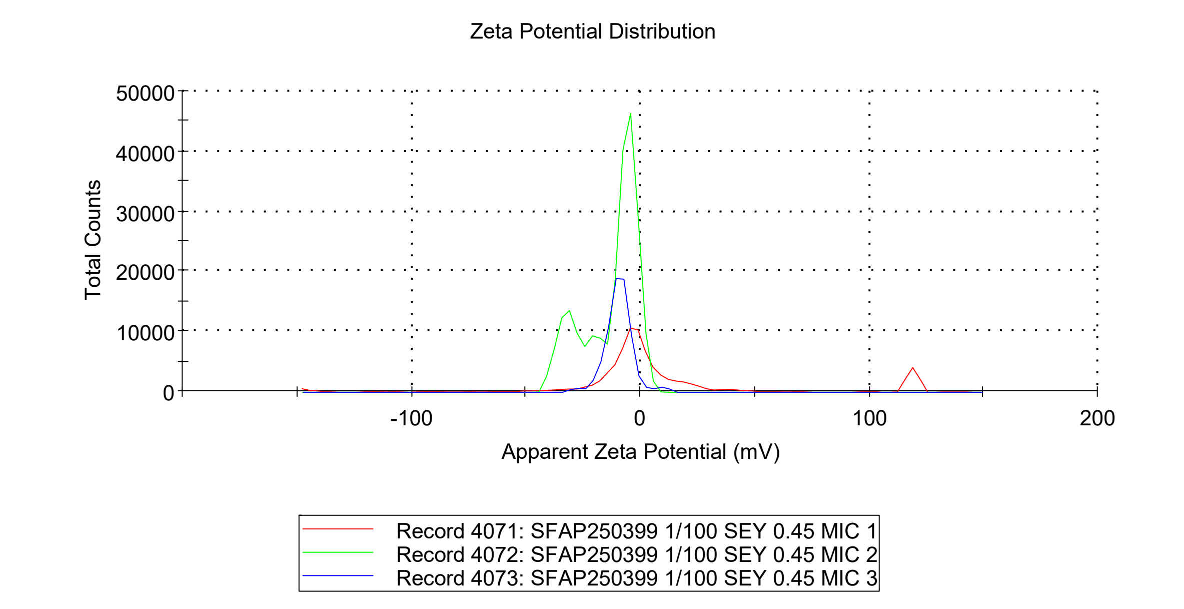

Supplement: Supplementary file 1 [file pharmaceutics-17-01434-s001.zip › pharmaceutics-3819368-supplementary.pdf]
